# Plants against cancer: the immune-boosting herbal microbiome: not of the plant, but in the plant. Basic concepts, introduction, and future resource for vaccine adjuvant discovery

**DOI:** 10.3389/fonc.2023.1180084

**Published:** 2023-07-31

**Authors:** Elizabeth Mazzio, Andrew Barnes, Ramesh Badisa, Stevie Council, Karam F. A. Soliman

**Affiliations:** ^1^ Divison of Pharmaceutical Sciences, College of Pharmacy and Pharmaceutical Sciences, Institute of Public Health, Florida A & M University, Tallahassee, FL, United States; ^2^ John Gnabre Science Research Institute, Baltimore, MD, United States

**Keywords:** edible microbiome, bugs as drugs, herbal microbiome, immune boosting, edible vaccine, immunotherapies, cancer, plant microbiome

## Abstract

The presence of microorganism communities (MOCs) comprised of bacteria, fungi, archaea, algae, protozoa, viruses, and the like, are ubiquitous in all living tissue, including plant and animal. MOCs play a significant role in establishing innate and acquired immunity, thereby influencing susceptibility and resistance to disease. This understanding has fostered substantial advancements in several fields such as agriculture, food science/safety, and the development of vaccines/adjuvants, which rely on administering inactivated-attenuated MOC pathogens. Historical evidence dating back to the 1800s, including reports by Drs Busch, Coley, and Fehleisen, suggested that acute febrile infection in response to “specific microbes” could trigger spontaneous tumor remission in humans. This discovery led to the purposeful administration of the same attenuated strains, known as “Coley’s toxin,” marking the onset of the first microbial (pathogen) associated molecular pattern (MAMPs or PAMPs)-based tumor immunotherapy, used clinically for over four decades. Today, these same MAMPS are consumed orally by billions of consumers around the globe, through “specific” mediums (immune boosting “herbal supplements”) as carriers of highly concentrated MOCs accrued in roots, barks, hulls, sea algae, and seeds. The American Herbal Products Association (AHPA) mandates microbial reduction in botanical product processing but does not necessitate the removal of dead MAMP laden microbial debris, which we ingest. Moreover, while existing research has focused on the immune-modulating role of plant phytochemicals, the actual immune-boosting properties might instead reside solely in the plant’s MOC MAMP laden biomass. This assertion is logical, considering that antigenic immune-provoking epitopes, not phytochemicals, are known to stimulate immune response. This review explores a neglected area of research regarding the immune-boosting effects of the herbal microbiome – a presence which is indirectly corroborated by various peripheral fields of study and poses a fundamental question: Given that food safety focuses on the elimination of harmful pathogens and crop science acknowledges the existence of plant microbiomes, what precisely are the immune effects of ingesting MAMPs of diverse structural composition and concentration, and where are these distributed in our botanicals? We will discuss the topic of concentrated edible MAMPs as acid and thermally stable motifs found in specific herbs and how these would activate cognate pattern recognition receptors (PPRs) in the upper gut-associated lymphoid tissue (GALT), including Peyer’s patches and the lamina propria, to boost antibody titers, CD8+ and CD4+ T cells, NK activity, hematopoiesis, and facilitating M2 to M1 macrophage phenotype transition in a similar manner as vaccines. This new knowledge could pave the way for developing bioreactor-grown/heat-inactivated MOC therapies to boost human immunity against infections and improve tumor surveillance.

## 1 Introduction

Microorganism communities (MOCs), encompassing bacteria, fungi, archaea, algae, protozoa, and viruses, often colloquially referred to as “bugs,” shape both innate and acquired immunity across all living systems, including plants, humans, and even malignant tumors, each of which hosts a unique microbiome. The rapidly evolving role of MOCs in cancer therapy, propelled by advances in metagenomics, has historical roots tracing back to the late 19th century. Physicians, including Busch, Coley, and Fehleisen, observed cancer patients undergoing remarkable spontaneous remission following acute febrile infections (38-40°C) induced by Streptococcus pyogenes (1, 2). These observations inspired collaborations and deliberate attempts to treat cancer patients with live MOCs, sometimes resulting in fatal sepsis. The sucessful approach involved the use of attenuated Streptococcus pyogenes (gram-positive) and Serratia marcescens (gram-negative), constituting the first microbial-associated molecular pattern (MAMP) tumor vaccine immunotherapy, comprised of toll-like receptor (TLRs 2,4) agonists bacterial LPS endotoxin/peptidoglycan (3). Coley’s tumor immunotherapy induced curative remission in approximately 50% of mesodermal embryonic origin cancers (sarcoma, lymphoma, leukemia, kidney, ovarian, and so on) for over 40 years (4). However, this approach was eventually supplanted by advancements in radiation, surgery, and immunosuppressive chemotherapies, which dominated the field of oncology for several decades (5, 6).

There is a resurgence of interest in tumor immunotherapies to achieve the same remission that Coley initially pursued over a century ago (5, 7, 8). However, these therapies must now overcome the immunosuppression imposed by mainstream chemotherapies and painkillers (e.g., cytostatic antineoplastics, opioids, and corticosteroids) (9–14), which destroy hematopoiesis *(*e.g., cytopenia, neutropenia), and lead to greater risk of infection, further aggravated by use of immunosuppressive antibiotics, and antipyretics (acetaminophen) (15). Infections, whether post-operative or otherwise, continue to be the leading cause of death in cancer patients (16–19). Moreover, the widespread use of synthetic drugs has had a cumulative negative impact on human immune intelligence in civilized societies. The human immune system relies on its capacity to amass a library of data on foreign pathogens, allowing it to eliminate recognized threats swiftly. Initial interaction often involves rapid infection resolution via fever and a robust immune defense.

Modern tendencies towards comfort over enduring sickness, and the overuse of antibiotics, antipyretics (Tylenol), and painkillers, have dulled immune intelligence. To add insult to injury, poor diet and pervasive societal stress, contribute to a widespread dysbiosis of the gut and host-immune dysfunction (effects transferable *in utero* from mother to child) (20, 21). The “hygiene hypothesis,” posing that minimal exposure to microbes in early life leads to dysbiosis, compounds the problem. This situation has been tied to epidemic non-communicable disorders and human cancers (22–24). Catering to sanitation and comfort is leading to an atrophy of human immune intelligence. As a result, there is an epidemic rise in chronic infections/inflammation which create refractory “T cell exhaustion” and a state of immune suppression, leading to greater susceptibility to various cancers (25–29). Conversely, nurturing immune intelligence through rigorous exposure to MOCs provides the routine cross-reactive targeting needed to destroy self-host malignant cells harboring similar tumor-associated antigens (TAAs) in a manner akin to infection response (30).

Immune suppression manifests itself as a loss of tumor immune surveillance, which is the controlling gateway for all human cancers to establish, develop, and thrive (31–34). In addition, immune suppression increases tumor initiation upon exposure to environmental carcinogens (e.g. pollutants, occupational fumes/dust, ionizing UV radiation (35)) carcinogenic microbes (36, 37), (*H pylori*), parasites (*O viverrine, C Sinensis*, and *S haematobium*) viruses (Epstein–Barr, human papilloma, hepatitis B, C, human herpes type-8, and human T-cell lymphotropic type-1) (38). These factors necessitate exploring new methods to enhance the immune system’s capacity to combat human cancers.

## 2 Plant medicine: the plant microbiome

**Figure 1:**
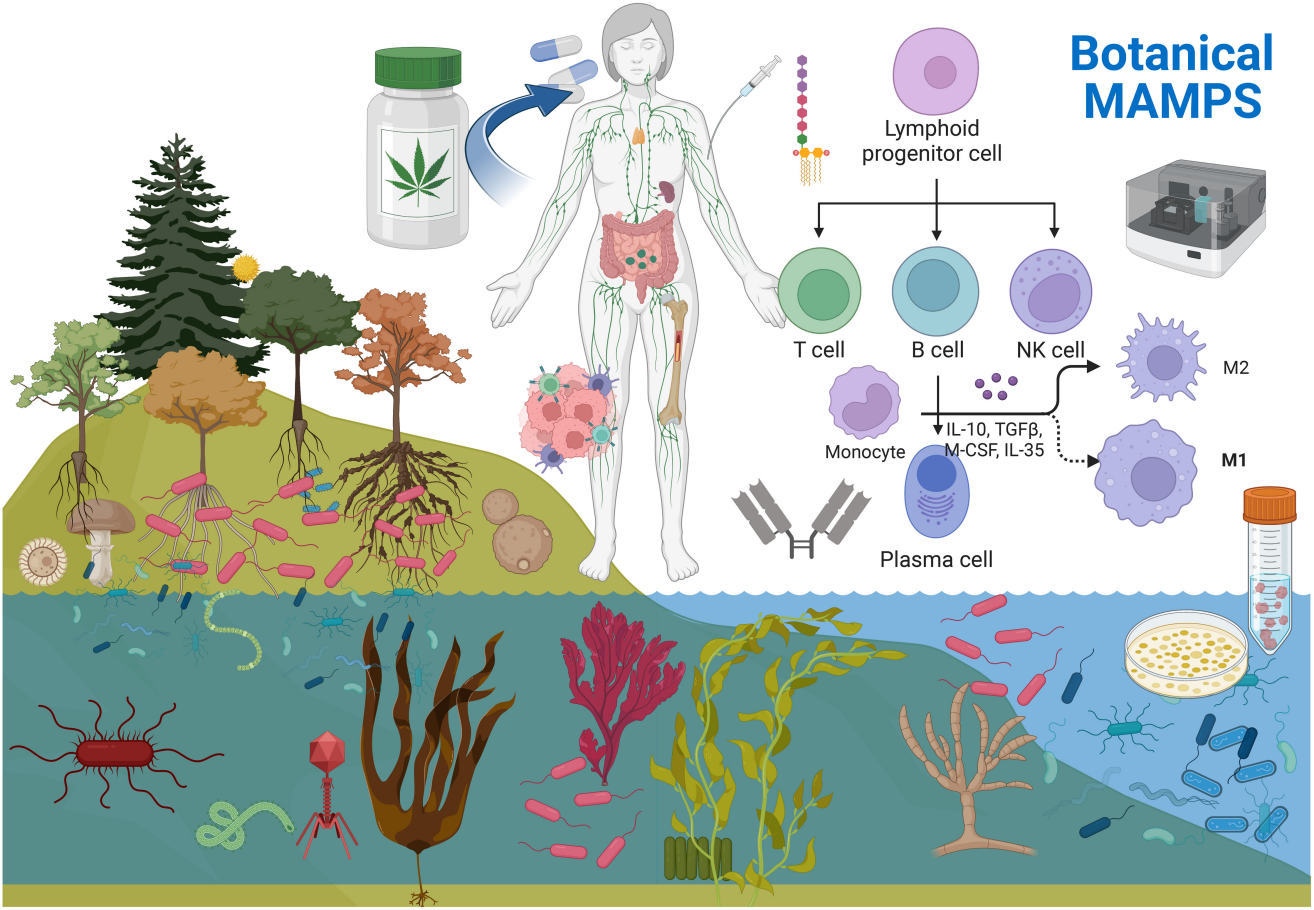
Microorganisms are diverse communities (MOCs) of bacteria, virus, fungus, protozoa etc. being ubiquitous in nature. MOCs are ever present within the richest reservoirs in roots, seeds, and sea algae’s. These MOCs end up in our foods, water and botanical medicines. Botanical products undergo post-harvest sterilization to ensure food safety, where MOCS (inactivated remain) . These MOCS are high in specific over the counter botanicals, few which are rich in MAMPs from gram negative bacteria. These TLR4 activating MAMPs have been inadvertently consumed for thousands of years, which can enhance innate and acquired immunity, while research efforts have been focused predominantly on the therapeutic effects of the plant/ phytochemical constituents. Given the plants microbiome is a unique component within/ but not of the plant - MOCS can be cultivated in a bioreactor / prior to inactivation and evaluated for effects on human health. Created with BioRender.com.

Immunological resilience is enhanced through the introduction of MOC antigens, a principle foundational to vaccines (39–43). That said, as of today there is meager research into the study of inactivated/attenuated plant microbiomes in our botanical medicines which contain product specific MOCs (including lethal pathogens) and how they impact human immunity. Plant-specific MOCs, are destroyed during food processing techniques like retort and pasteurization. However, not all MOCs leave a trail of immune provoking microbiome-associated molecular patterns (MAMPs), and their ubiquity varies, issues discussed below. Edible, immune-provoking MAMPs are nature’s concentrated reservoirs of immune stimulants within the plant world. **Figure 1** MOCs are ever present as controlling elements in plant health, growth, maturation, and ecological control whereby numerous factors influence the diversity and concentration. These include the plant’s/part phytochemical profile (e.g., anti-biotic, anti-fungal properties), its location and part (e.g., above-ground stems/leaves exposed to UV sterilizing sunlight and rainwater wash, or subsurface parts exposed to different gas compositions and minerals), the plant’s tactile nature (e.g., alginates), MOCs altered during maturity at harvest, and complex interactions during growth, among others. All medicinal herbs and spices contain MOCs, where food safety regulations by the American Herbal products association (AHPA) and National Science Foundation/American National Standards Institute (NSF/ANSI) (44–47) require microbial reduction and third-party testing to establish 1) an absence of viable pathogens and mycotoxins, 2) a residual threshold yield of live non-pathogenic coliform, aerobic plate counts, total yeast & molds reported as colony forming units (CFU)/g (44–49). However, our understanding of which plant microbiomes are immune-boosting and how their residual trail of microbiome/MAMPs impacts health, from pathogenic to non-pathogenic, remains limited. Edible MAMPs, often acid and thermally stable, can act on pattern recognition receptors (PRRs) in the upper gut-associated lymphoid tissue (GALT), Peyer’s patches, and lamina propria, influencing innate and acquired immune systems (44–47, 50). Yet, despite their ubiquity in foods, most studies on the “edible plant microbiome” focus on identifying the taxonomy of live microorganisms present, with little consideration of their potential health implications (51–58).

Humans have consumed plant medicines and their resident MOCs for millennia, attributing the health benefits only to the plant and constituent phytochemicals. However, a small number of research teams have proposed that the health benefits of certain herbs may instead be due to their inactivated microbial biomass after separating the plants bioactive MAMPs from the plant itself (39–41). This theory aligns with the broader field of microbiomic science and the simple logic that the immune system responds aggressively not to phytochemicals or drugs but to the presence of foreign antigenic microorganism debris (MAMPs), the basis for vaccine and adjuvant development. A few research teams have discovered that MOC-MAMP PRR TLR4 agonists are responsible for immune boosting effects in the following herbs; Black walnut, Echinacea Root, Ginseng Root, Alfalfa Seeds (59) Astragalus Root (60, 61), Angelica Root (62), and Wheat, the latter who introduces the concept of edible LPS found in wheat from *Pantoea agglomerans* as a “modern-day nontoxic Coleys toxin” with limitless capacity when taken orally (63–66). Our recent research corroborates the findings of all of these studies, indicating the immune boosting properties are not of the plant, but rather attributable to its MAMP MOC biomass, while we now expand this list to include 65 out of approximately 2000 OTC products tested containing TLR4 PRR agonists (Publication Pending, Mazzio et al., 2023. Journal of Funcitonal Foods ).

Our research findings directly align with ecological studies reporting similar soil-embedded rhizome microbiomes (67) (e.g., echinacea, stinging nettle, burdock etc.) and high concentrations of gram-negative microbes and biofilms in edible sea kelps and other marine vegetation, including *Granulosicoccus antarcticus*, *hellea balneolensis (Gammaproteobacteria) bacteroidetes and Alphaproteobacteria*, these including *Fucus vesiculosus, N. leutkeana* (Bladderwrack), *P. scouleri* (Surf Grass), *Laminaria setchellii* (Kelp) *Chondrus crispus* (Irish Moss), *Laminaria ochroleuca* (Kelp/Brown Seaweed)*, Palmaria palmate* (Dulse), etc. (68, 69).

According to our research, approximately 98% of herbs, fruits, and vegetables do not contain bioactive toll-like receptor (TLR4) agonists, which suggests that immune-modulating microbiomes in plants and herbs are not ubiquitous but instead result from natural forces that encourage their accumulation.

### 2.1 Diverting research focal points

Significant research has been directed toward peripheral topics, often overlooking the potential human health aspect of ingesting inactivated MOCs. None the less, the concept that ingestion of “dead bugs” can impact human health is gradually gaining acceptance, as seen in the emerging fields of para-probiotics (inactivated probiotics) (70–72) development of oral vaccines (73, 74) and with greater understanding as to the anatomy of the human gut microbiome and its PRRs (44–47, 50, 75). Despite this, most of the work carried out on the human microbiome to date has focused on the “living microbiome” (probiotics, prebiotics, and microbiota cultures) as symbionts/pathobionts (76–79). Meanwhile, therapeutic research in plant medicine continues to focus on plant phytochemicals rather than the plant’s inactivated microbiome. Plants contain a diverse array of “phytochemicals” such as flavonoids, phenolics, alkaloids, glycosides, lignans, and triterpenoids, which exhibit a broad range of protective properties, including anti-inflammatory, analgesic, antipyretic, antimalarial, antibacterial, antiprotozoal, antioxidant, antifungal, and antiviral effects (80). Inadvertently, the antimicrobial capabilities of these plant chemicals have been exploited for centuries for the preservation and treatment of infections. Today, the predominant focus on anti-cancer natural medicines, also continues to be on the phytochemicals. Thousands of studies demonstrate phytochemicals to manipulate cellular signaling pathways such as apoptosis (Bcl-2/Bax), oncogene transcription, gene induction, enzyme expression/activity (e.g., topoisomerase, cyclooxygenase, matrix metalloproteases), cell cycle regulation, and modulation of signaling systems associated with rapid tumor growth (e.g., MAPK/ERK pathway, PI3K/AKT/mTORC1), or to activate DNA repair mechanisms (80–85). A similar emphasis on chemicals is observed in research exploring the interaction between microbes and cancer, specifically focusing on secondary metabolites produced by *Actinobacteria* and *Streptomyces* spp. These metabolites, which include polyphenols, indolocarbazoles, anthracyclines, halogenated compounds, polyketides, anthracenes, and alkaloids, form the foundation of traditional chemotherapy drugs like doxorubicin, mithramycin, and mitomycin C (86).

While plant chemicals can prevent cancer by attenuating chronic inflammation that initiates cancer (27, 87), managing already-established cancer requires a robust and sharp immune boost, necessitating a pro-inflammatory response.

### 2.2 MAMPS in anti-tumor immune therapies

The potential to boost the immune system to overpower and destroy a human tumor forms the foundation for future immunotherapy breakthroughs. All tumor immune therapies share a common goal: to “boost,” “activate,” or “reawaken” a dormant host immune system subdued by the tumor’s immunosuppressive barrier to regain control over the mechanisms capable of destroying malignant cells (5, 7, 8); this requires a pro-inflammatory (immune-boosting) response, not an anti-inflammatory response. Therapies (similar to Coley) have thus far explored combination of synthetic hyperthermia (fever) ± inactivated microbes containing potent MAMPS (6, 88, 89), MAMP vaccines (90, 91), exogenous cytokines (IL-2, 15), interferon-alpha (IFNα) or granulocyte-macrophage colony-stimulating factor (GM-CSF)), adoptive T cell therapies with direct targeting of (tumor-associated (TAA)/tumor-specific antigens (TSA)), checkpoint inhibitors which remove/braking systems (mAb), ipilimumab, nivolumab, pembrolizumab, etc.) (92–95) or autologous/allogenic tumor vaccines primed with strong immunological bacterial laden adjuvants and co-stimulatory cytokines (96).

Pharmacodynamically and mechanistically edible MAMPS may do the same, having direct access to the immune system through the gut mucosa. The upper GI can capture (by mucin), identify, respond to, and build an acquired immunity database to foreign antigens while offering 15 times the surface area than the large intestine (97). The upper GI also houses plentiful GALT-/LP PRRs, in epithelial intestinal and immune cells (neutrophils, macrophages/monocytes, dendritic cells, mast cells, T and B lymphocytes), and a highly integrated signaling surface readily acted upon by MAMPS on C-type lectin-like receptors (CLRs) (Dectins, Mincle, and Mcl), nucleotide-binding oligomerization domain (NOD)-like receptors (NLRs), and Toll-like receptors (TLRs) (98). As for TLRs, these are housed in the gut immune plexus on either cell surfaces (TLR1, 2, 4, 5, 6) or/in (endosomes TLR3, 7, 8, 9) (99, 100). Known edible MAMPS that would activate these include food-based bacterial LPS, lipoproteins, zymosan, byglycans, acylated lipopeptides, lipoteichoic acid, peptidoglycans, modulin, dsRNA from bacteria, glycolipids, fibrinogen/fibronectin, heat shock proteins, uric acid, flagellin (TLR5), ssRNA of microbial origin and unmethylated CpG rich DNA (101–104).

Just in the case of TLR agonists alone, these are widely in our foods and vaccines as adjuvants, being pro-inflammatory, with a capacity to boost antibody titers, CD8+ and CD4+ T cells, cytokine levels (IL-12, TNF-α, IFN-γ, IL-6 and type I interferon), chemokines (monocyte chemoattractant protein-1 (MCP-1/CCL2), macrophage inflammatory proteins (MIP-α/1-β) MyD88, IL-1R), and related signaling pathways (IRAK, MAPK) through transcriptional activation of NF-κB and AP-1 (42, 43). PRR TLR adjuvants used in vaccines include agonists of TLR9 (CpG oligodeoxynucleotides (ODN)) - the hepatitis B virus vaccine Heplisav-B, MGN1703 for cancer vaccines; TLR3 (poly-IC/ICLC derivates (Ampligen® Hiltonol®)) for cancer and HIV vaccines, TLR4 (monophosphoryl lipid A derivative agonist AS04, for Cervarix (human HPV)/Hep B (Fendrix®), AS01 for the herpes zoster vaccine Shingrix®, glucopyranosyl Lipid A for influenza vaccines), TLR 5 agonists (flagellin derivatives such as Mobilan or entolimod) or TLR7 (resiquimod (an imidazoquinoline) employed with cancer vaccines (42).

While dead bugs in our botanical medicines have not yet been explored in large, fungal molecules are subject to exhaustive research, as in the case of the peptide fragment zymosan; also known as β-glucan derived from *Saccharomyces cerevisiae* (105–112). When ingested orally, β-glucans activate (M-cells)/, Peyer’s patches and boost innate and systemic acquired intelligence (107–109). This mechanism of action has direct relevance to tumor immunotherapies given it can elicit sharp responses which foster 1) macrophage phenotype polarization in tumor-associated macrophages (TAMs) to M1 anti-tumor fighter macrophages (110, 111), 2) reduce the load of tumor immune suppressors such as myeloid-derived suppressor cells (MDSC) or Tregs (112) 3) heighten activated CD4(+), CD8(+) T cells and the IL-2 IFN-γ response (113) 4) augment hematopoiesis (106) prompt maturation of neutrophils, macrophages, dendritic cells, and NK cells (Dectin/TLR/complement receptor 3 (CR3), CD11b CD18) (114, 115) and 5) initiate iC3b-opsonized targeting of tumor cells for phagocytosis and degranulation (106, 107, 114, 116), linked to reduced tumor burden in mammals (105, 114, 115, 117–119).

Using edible immune boosters such as zymosan could synergize the efficacy of checkpoint inhibitors, whereby taking the foot off the tumor immunosuppressive barrier (immune checkpoint inhibitors) combined with the second foot on acceleration “immune boosting” would provide therapeutic advantage. (115, 120, 121). Edible MAMPS (zymogen or other) reaching the GI would evoke the transfer of enemy antigenic data through M cells to antigen-presenting cells (APCs), which then travel to the mesenteric lymph node (122) concomitant to lamina propria APC dendritic cell branching to systemic lymphatic vessels (75, 123–125) evoking T cell clonal expansion (126–128). Moreover, oral administration of MAMPS, like LPS, may enter into blood circulation through lipid absorption or chylomicrons by intestinal epithelial cells (50) triggering systemic innate and acquired immune system response as evidenced in mammals (75).

### 2.3 Are edible PRR-TLR4 agonists endotoxin?

Are we suggesting that edible endotoxin can alter immunity? Yes, the practice of consuming endotoxin has been applied for hundreds - thousands of years, before the discovery of microbes in cultural medicine for example edible spirulina, chlorella, sea moss, and sea kelps. There is enormous confusion surrounding the term “endotoxin” because it is a misnomer. The term “endotoxin” today is an open-ended, vague term to describe *all* gram-negative microbial cell wall LPS with the lipid A the “ toxin” all alive or dead, all pathogenic or non-pathogenic, and all-inclusive to every type of taxonomic and cell wall polysaccharide variation. The term endotoxin is one term, used to ascribe deadly infectious (sepsis) with multi-organ failure and death, to health products sold OTC taken by billions of consumers across the globe every day, e.g., as previously stated: LPS *Arthrospira platensis* (Spirulina). Endotoxins are in many cases endo-immunomodulators (not toxins) with beneficial effects justifying their use as peptide-based anti-cancer vaccines adjuvants (101). Many studies show endotoxin in immune-competent cancer models can reverse the negative effects of chemotherapy and shrink/sometimes eradicate human tumors in a manner as described by Coley (129–138) with the ability to boost NK cell activity, activate macrophages and stimulate hematopoiesis of lymphocytes (139–155). And, if MAMP-laden edible products bear resemblance to Coley’s toxin (156), then as longitudinal fibrils contiguous (157) they too could very likely evoke mitogenic B cell activation (158) influence the complement system (156), stimulate macrophages to lyse tumor cells (159) and bestow all benefits known to LPS/TLR4 agonists and their use in anti-cancer medicines when taken orally (63, 65, 66).

This concept is substantiated further by, numerous animal and human studies demonstrating that exposure to LPS in early life can stimulate systemic immunity, being inversely related to the occurrence of hay fever, atopic asthma, and atopic sensitization (160). Moreover, LPS, a standard endotoxin, has been identified as a vital immune system stimulant (161). Specifically, LPS derived from Escherichia coli has been proven to elicit a robust immune response via TLR4 signaling; this, in turn, triggers the release of pro-inflammatory cytokines, leading to an enhanced immune response (162). Immunotherapies that leverage LPS have shown promise in preclinical and clinical studies. For instance, MPL (monophosphoryl lipid A), a derivative of LPS, has been successfully used as an adjuvant in anti-cancer vaccines (162, 163). Despite these advancements, the potential of dead bugs, especially their endotoxins, in therapeutics and immunotherapies remains largely unexplored. The primary barrier has been the conventional perception of endotoxins as harmful substances associated with diseases such as sepsis. Therefore, the potential of dead bugs in therapeutics and immunotherapies warrants further exploration. Future studies should focus on unraveling the specific immune-modulatory mechanisms of endotoxins and other components of dead bugs and their potential applications in treating diseases such as cancer. With technological advancements and an understanding of the gut microbiome, the prospect of leveraging dead bugs in therapeutics is promising.

## 3 LPS/TRL4 agonists and cancer models

If the administration of gram-negative (non-strain specific) LPS can reduce tumor burden, then why is the literature riddled with contradiction? These conflicting reports appear consistent when organized by model type and route of administration. For example, pro-tumor effects of LPS are consistently reported in (immune-deficient) animal models, or isolated cancer cells *in vitro* (without an immune coculture) or by assumptions drawn on greater expression levels for TLR4 reported in cancer vs. healthy adjacent tissue (164–175). However, anti-tumor effects of LPS/TLR4 agonists are consistently reported in fully immune competent animals, the majority showing greater survival time, curative remission in a significant % of test groups similar to Coley’s toxin, reduction of adverse effects of chemotherapy and radiation, including resistance (130–133, 176–178), and effects augmented when combined with anti-tumor cytokines (e.g. G-CSF) (134). LPS administration, when injected directly into tumors (immune-competent models) - elicits massive macrophage and neutrophil trafficking (135) evoking nearly complete and total tumor regression consistently reported (136, 137). In humans, oral administration of LPS from wheat (*Pantoea agglomerans*) shows recovery and remission in 62% of cancer patients (138), whereas other sources (e.g., LPS *Alistipes shahii*) can achieve a reduction in side effects associated with radiation/chemotherapies (129).

### 3.1 LPS/TLR4 agonists: mechanism of action

**Figure 2:**
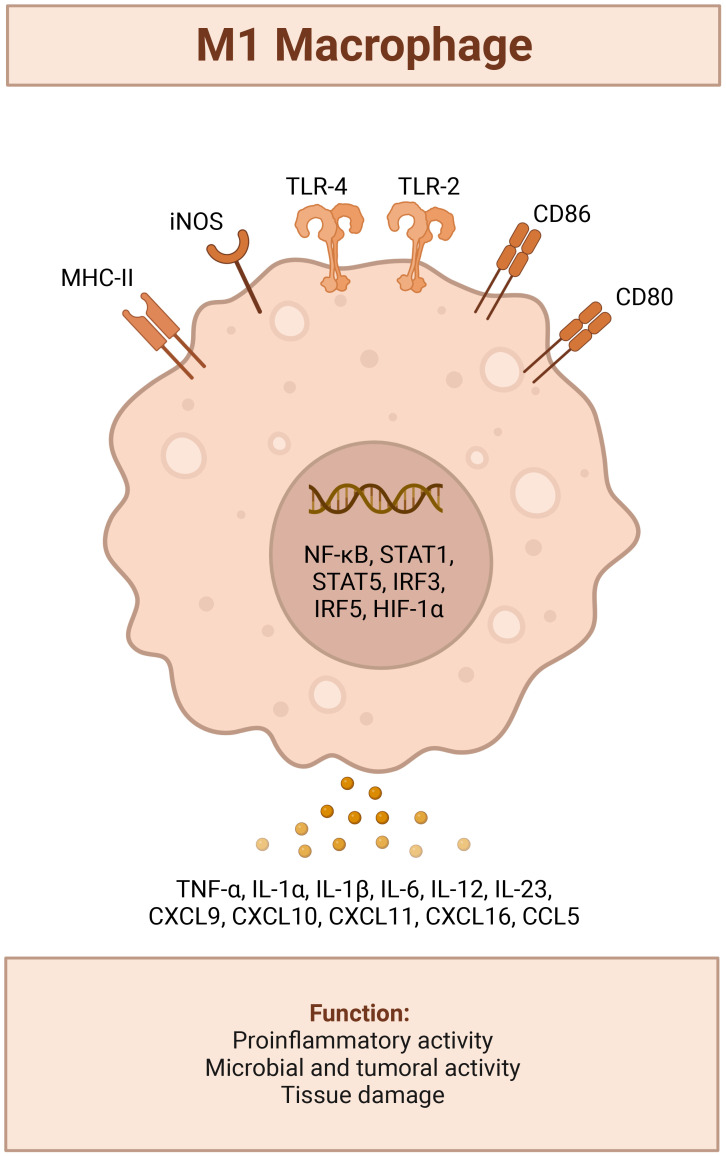
LPS TLR4 activated M1 tumor suppressor Macrophage Phenotype; triggers recruitment of MyD88 to the cytosolic domain, activates mitogen activated protein kinase signaling to elicit translocation of nuclear factor kappa-light-chain enhancer of activated B cells (NF-κB) and upregulation a host of genes involved with leukocyte recruitment (CCL2,CCL6,CCL12, CXCL10, CXCL11,CXCL12,CXCL13), a pro-inflammatory response (IFN-γ, TNF –α, IL-6 or Type I interferons IFN-α and IFN-β), iNOS induction and production of NO2.[(42)] Created with BioRender.com

The mechanism by which LPS reduces tumor size remains a topic of continued research, but it is generally thought to be closely tied to its critical role in regulating macrophages. Several steps mediate its anti-tumor effects: 1) LPS first binds to the LPS first binds to protein/sensitizer CD14 (179) 2), both then binding to the TLR4/MD-2 complex (180), 3) which triggers MyD88 signaling, 4) and leads to an IFN-type 1 response, NF-kappaB and activation of TAMs in the TME, thus switching “on” the M1 anti-cancer fighter phenotype (pro-inflammatory, anti-tumorigenic) and overcoming the acquiescent M2 (anti-inflammatory pro-tumorigenic phenotype) (7, 181, 182). **Figure 2** In immune-competent animals, the transition from M2 to M1 TAM phenotype is associated with the re-awakening of T-cell mediated adaptive host immune response that recognizes malignant cells However, this transition’s mechanisms are still poorly understood (101, 183–186). M1 TAM phenotypes coincide with increased activation of CD4(+) and CD8(+) T cytotoxic cells, NK cells, higher IFN-gamma, reduction in myeloid-derived suppressor cells, and Tregs, all of which contribute to reducing tumor burden (186–188). These responses are also directly related to the functional relay of TLR4/Myd88 receptor adaptor response (133, 189, 190). For instance, TLR4−/− mice exhibit rapid tumor growth following inoculation (191) which corresponds to a deficiency in CD8+ CD4+ T and activated NK cells (42, 192).

The factors that maintain the M2 phenotype are still debated, but reportedly involve continuous exposure to cytokines released from the stroma/tumor itself, such as suppressive E-receptor factor, hyaluronan (193–196) or the dominance of gram-negative bacteria in the ‘tumor microbiome,’ as seen in gemcitabine-resistant pancreatic ductal carcinoma (197). The future therapeutic use of LPS/TLR4 agonists could enhance the efficacy of immune checkpoint inhibitors (ICIs) (198–201), increase the effectiveness of monoclonal antibodies (MAbs) (e.g., trastuzumab) (202) and boost the potency of platinum- and taxol-based chemotherapies (203). As of now, TLR4 agonists are primarily used as adjuvants in tumor vaccines (204–207) and in adoptive anti-tumor immunotherapies (208) as well as in dendritic cell-based therapies specific to tumor antigens (209).

### 3.2 The tumor microbiome: an immune suppressive barrier

The discovery of the tumor microbiome has brought significant challenges to the conceptual basis of tumor vaccines. This discovery creates a model akin to a house within a house. The larger house represents the human microbiome’s role in regulating systemic immunity, while the smaller house symbolizes the tumor microbiome’s role in controlling its local environment. The host microbiome, which largely colonizes the mucosal tissue of the oral/nasal cavity and gastrointestinal, vaginal, and urogenital tracts (97, 210), is sensitive to environmental factors (e.g., drugs, including antibiotics and chemotherapy, prebiotics, probiotics, alcohol, diet, stress aging, and exercise) (211–213).

When the normal flora is overtaken by pathogenic flora, as documented in thousands of studies [indicating changes in the composition of microbial communities in malignant versus healthy non-malignant tissues], it increases the risk of cancer (76, 214–216). Pathogenic flora that contribute to cancer can spread locally (e.g., from the vagina to the cervix (217), or from the respiratory system to the lungs (218)) or distally as bacteria originating in the oral cavity (e*.g., Porphyromonas gingivalis, Tannerella forsythia, Veillonella parvula, F. nucleatum, Parvimonas Micra*, etc.), have been found associated with cancers of the esophagus (219), GI/colon (220) pancreas (221) and liver (87). Once cancer is established, microorganisms can infiltrate the tumor microenvironment (TME), working synergistically with tumor-infiltrated leukocyte subpopulations (LSPs); tumor-associated macrophages (TAMs), tumor-associated neutrophils (TANs), myeloid-derived suppressor cells (MDSCs), CD4+CD25+Foxp3+Tregs creating a robust and strong immunosuppressive barrier (37, 87, 222, 223).

Overcoming the immunosuppressive barrier circumscribing tumors can be achieved by either the complete obliteration of the tumor microbiome, which can restore immune competence, decrease immune suppressors (MDSCs), and enhance Th1-type CD4+/cytotoxic CD8+ T function (221) or boosting the immune system (using immunotherapies), similar to the original MAMP vaccine by Coley, but with greater specificity (90, 224).

## 4 MAMPS and chronic inflammation

There is a potential double-edged sword involving the biological effects of LPS/TLR agonists, which on the one hand may boost immune response, and on the other hand are used in experimental models of inflammatory oxidative injury. Generally, repeated administration of LPS in animals is an established model of inflammatory injury to assess the value of anti-inflammatory agents (225, 226). The understudy of the paradox between immune stimulating vs chronic inflammation is a needed area of research given the large number of individuals, globally, who consume daily supplements containing fungal yeast (b-glucan)/microbial MAMP TLR agonists. There are several major plausible outcomes of repeated daily use 1) the establishment of biological tolerance, which can provide resilience to allergies (e.g., pollen) (227), eczema (228), or osteoporosis (229), infection and cancer (230, 231) or 2) chronic inflammation (232, 233) leading to refractory T-cell exhaustion, upregulation of (PD-1)/(PD-L1) axis, MDSCs and enhanced capacity of carcinogen-mediated tumorigenesis (26, 27, 234, 235);this raises an important question: Could repeated oral administration of MAMP-rich herbs like sea moss/kelp and roots result in immune suppression due to chronic inflammation, either of which could potentially initiate human cancers? In this context, promoting the intake of anti-inflammatory phytochemicals might be advisable to reduce the risk of cancer initiation (28, 236). By contrast, repeated TLR-4 stimulation could be linked to chronic infections and inflammatory conditions and may be contraindicated for individuals with autoimmune diseases (237). These are pressing issues that require further investigation for clarity.

## 5 Conclusion

This review looks into the potential of heat inactivated MOCs, particularly in botanicals, to boost immunity and improve illness outcomes. Specific immune-boosting herbs are abundant with dead microbes, thereby holding significance for drug discovery endeavors to develop oral, edible tumor immunotherapies. Future research should explore the intriguing hypothesis that the health benefits of certain herbs may be due to their inactivated microbial biomass rather than the plant’s phytochemicals; this would not only align with the broader field of microbiome science but also with the simple logic that the immune system reacts to foreign antigenic microorganism debris (MAMPs), the basis for vaccine and adjuvant development. Despite our growing knowledge, significant gaps still need to be discovered. For example, current research primarily focusing on cataloging the live microorganisms in the “edible or medicinal plant microbiome,” are often without considering potential health implications. While we understand which plant microbiomes survive food safety regulations, we need comprehensive knowledge of MAMPS from inactivated MOCs and their immune-boosting potential, including the concentrations and taxonomy, from pathogenic to non-pathogenic.

This potential paradigm shift could have significant implications for our understanding of plant-based health and for developing new therapeutic approaches. Furthermore, the historical backdrop of MOCs’ influence on immunity, as indicated by cases of spontaneous tumor remission due to acute febrile illnesses, lays the groundwork for investigating MOCs’ therapeutic potential in cancer immunotherapy. This has the potential to transform our approach to disease prevention and treatment, specifically by using the ability of MOCs to strengthen human defense against pathogens and improve tumor surveillance.

## Author contributions

All authors listed have made a substantial, direct, and intellectual contribution to the work, and approved it for publication.
